# Effects of prenatal early food and micronutrient supplementations on body size and cardiometabolic risk factors in the 15-year-old offspring – the MINIMat randomised trial

**DOI:** 10.1016/j.lansea.2026.100878

**Published:** 2026-10-05

**Authors:** Eero Kajantie, Rubhana Raqib, Eva-Charlotte Ekström, Monjur Rahman, Pieta Näsänen-Gilmore, Syed Moshfiqur Rahman, Jesmin Pervin, Mohammad Redwanul Islam, Shams El Arifeen, Maria Kippler, Anisur Rahman

**Affiliations:** a Clinical Medicine Research Unit, MRC Oulu, University of Oulu and Oulu University Hospital, PL 9000, 90014, Oulun yliopisto, Oulu, Finland; b Welfare Epidemiology and Monitoring Unit, Finnish Institute for Health and Welfare, PL 30, 00271, Helsinki, Finland; c International Centre for Diarrhoeal Disease Research, Bangladesh (icddr,b), GPO Box 128, Dhaka, 1000, Bangladesh; d Department of Women's and Children's Health, Global Health and Migration Unit, Uppsala University, Akademiska sjukhuset, SE-751 85, Uppsala, Sweden; e Data Operations Management Team, Finnish Institute for Health and Welfare, PL 30, 00271, Helsinki, Finland; f Institute of Environmental Medicine, Karolinska Institutet, SE-17177, Stockholm, Sweden

**Keywords:** Cardiovascular, Cardiometabolic, Protein, Energy, Pregnancy, Pregnant

## Abstract

**Background:**

Evidence on the effects of maternal nutrition during pregnancy on offspring cardiometabolic risk is mixed. We hypothesized that 15-year-olds, whose mothers were randomised to maternal early pregnancy protein-energy supplementation, are taller and have lower low-density lipoprotein cholesterol than controls. We also assessed effects of prenatal multiple micronutrient supplementation (MMS) on adolescent body size and cardiometabolic risk factors.

**Methods:**

In MINIMat trial (Maternal and Infant Nutrition Interventions in Matlab; ISRCTN16581394) in rural Bangladesh, pregnant women were randomised in a 2:3 factorial design to early invitation to food supplementation (around gestational week 9), vs standard programme (usual invitation); MMS was compared with folic acid and iron. Of 3267 singletons with valid birth weight, 2300 (70.4%) were measured height and weight, plasma cardiometabolic biomarkers and blood pressure at 15 years, between September 2017 and June 2019. We analysed data by linear and logistic regression, adjusting for sex, age and prenatal confounders.

**Findings:**

Adolescents whose mothers received early invitation had 0.08 units higher height z score (95% CI 0.01, 0.16) and less of them were stunted (<−2 SD; 17.4% vs 21.4%, risk difference 4.1%; 95% CI 0.8%, 7.3%; OR 0.77; 0.63, 0.95) than usual invitation group. Early invitation and MMS were unrelated to BMI, plasma cardiometabolic biomarkers or blood pressure.

**Interpretation:**

The effect of early prenatal food supplementation on height persists at 15 years. We found no effect of this intervention or MMS on cardiometabolic factors.

**Funding:**

Swedish Research Council, Finnish Research Council, Finska Läkaresällskapet, Pediatric Research, Novo Nordisk, Gyllenberg and Jusélius Foundations.

**Research in context**

**Evidence before this study**

We searched PubMed from 1 January 1980 to 29 May 2026, without language restrictions, for follow-up studies based on randomised controlled trials evaluating the effects of maternal protein-energy supplementation or multiple micronutrient supplementation during pregnancy on offspring body size or cardiometabolic risk factors in childhood (aged four years or more), adolescence or adulthood. We also searched for lists of references in identified papers. We found 16 published papers based on eight trials. Four of them report on maternal protein-energy supplementation. Of these, three were cluster-randomised: one conducted in Guatemala assessed at 21–29 y, one in The Gambia at 11–17 y and one in India at 13–18 y. The India trial also included protein-energy supplementation for the child. The search also identified an earlier 4.5 y follow-up of the individually randomised MINIMat trial in Bangladesh which we now report on.

Two of the trials, those in India and Bangladesh, aimed to identify pregnancies and start supplementation as early as possible. These trials showed taller height in children whose mothers received the intervention, whereas the trial in The Gambia, where the supplementation started around week 20, indicated marginally shorter height. Effects on cardiometabolic risk factors were mixed: the lower total and HDL cholesterol in the MINIMat study at 4.5 years was not seen in other studies, but two of these suggested more favourable glucose metabolism indicators.

The five trials on multiple micronutrient supplementation included two trials in Nepal, one cluster-randomised assessed at 6–8 y and one individually randomised, assessed at 8.5 y. Further, there was one individually randomised trial in India assessed at 5–10 y, a cluster-randomised trial in China assessed at 10–14 y and an earlier 4.5-year follow-up of the present individually randomised MINIMat trial in Bangladesh. The Chinese trial found lower blood pressure among those whose mothers received multiple micronutrients. There was no evidence on an effect of multiple micronutrient supplementation on body size or cardiometabolic biomarkers, although no study assessing these outcomes was followed up after 10 years.

**Added value of this study**

Children whose mothers were randomised to receive early (first trimester) invitation to food supplementation remain taller and less likely to be stunted at 15 years, compared with children whose mothers received food supplementation at their liking in mid-pregnancy. We were unable to see any effects on lipid profile, other cardiometabolic biomarkers or blood pressure. Multiple micronutrient supplementation during pregnancy was unrelated to body size or cardiometabolic risk factors at 15 years.

**Implications of all the available evidence**

While the observed effect on adolescent height and stunting is modest, the finding serves as a proof of principle that the effects of food supplementation early in pregnancy on body size persist at least to adolescence. We were unable to see any effect on cardiometabolic risk factors suggested by previous evidence. However, overall levels of these factors was low and future follow-up is needed to assess possible effects that might arise at later ages. Our finding reinforces previous findings of no effect of multiple micronutrient supplementation on body size or cardiometabolic risk factors in childhood and extends them to adolescence.

## Introduction

The Developmental Origins of Health and Disease (DOHaD) theory suggests that factors acting during sensitive fetal and childhood periods have lifelong effects on the function of the organism and accordingly risk of disease.^1^^,^^2^ The theory is supported by two main categories of evidence. First, an overwhelming body of observational studies link markers of fetal life adversities, such as birth weight, with lifelong health.^1^^,^^3^, ^4^, ^5^ Second, experimental studies in animals prove causality and uncover underlying mechanisms.^1^ DOHaD theory has gradually become an essential part of mainstream medical science. Yet, there is still little experimental evidence in humans that would prove causality and provide evidence basis for policy actions to promote lifelong health by specific interventions in early life.

Randomised trials of nutritional interventions during pregnancy provide a useful setting to obtain such evidence. Many of these trials have been conducted in undernourished populations where common interventions include energy-protein^6^ or multiple micronutrient^7^ supplementation, or both, and primary outcomes commonly include birth weight, infant mortality, and infant growth and development. Some of the intervention cohorts have then been followed up to study cardiometabolic risk factors, but the results have been mixed. There are observations of small favourable effects of protein-energy supplementation on glucose metabolism,^8^, ^9^, ^10^ lipid profile^11^^,^^12^ or linear growth^9^ in some studies that are not seen in others.^9^^,^^10^^,^^13^ While some studies have suggested favourable effects of multiple micronutrient supplementation on some cardiometabolic risk factors,^12^^,^^14^^,^^15^ others have shown no effects^16^^,^^17^ or even adverse risk indicators,^12^^,^^18^ and the effects have generally not been replicated or present in meta-analyses.^19^^,^^20^ Generally previous studies have not been powered to assess long-term childhood effects of protein-energy or multiple micronutrient supplementation during pregnancy, mostly including no more than slightly over 1000 participants, and heterogeneity in the interventions have limited the use of meta-analytic approaches. Thus the effect of these interventions on body size and cardiometabolic risk factors in childhood and adolescence remains poorly known.

In 2001–2003, the MINIMat (Maternal and Infant Nutrition Interventions in Matlab) trial in Bangladesh randomised pregnant women in a 2 × 3 factorial design to receive early invitation to food supplementation (around week 9 of gestation), vs usual invitation to food supplementation (start at the women's own liking usually around week 20), together with comparisons of multiple micronutrient supplementation, with folic acid and two doses of iron (30 and 60 mg).^21^^,^^22^ The early invitation to prenatal food supplementation in combination with multiple micronutrients resulted in markedly lower infant and under-five mortality but had no effect on the other primary outcomes, maternal hemoglobin and birth weight.^22^ The cohort of children born in the trial has since then been followed up. Between birth and 4.5 years, children whose mothers had received early invitation to food supplementation were on average 0.08 SD taller and less likely to be stunted than those whose mothers received usual invitation.^23^ At 4.5 y, the early invitation group showed lower total and low-density lipoprotein (LDL) cholesterol and apolipoprotein B concentrations^12^ and marginally lower diastolic blood pressure.^24^ Multiple micronutrient supplementation showed a more mixed pattern of findings.^23^, ^24^, ^25^, ^26^

The MINIMat children have now become adolescents, and we invited them to a new clinical assessment. Based on the findings at 4.5 y and tracking of plasma lipids across childhood,^27^ our primary hypothesis was that the effects of early invitation to food supplementation on height and plasma lipid profile would be present at 15 y. We also assessed the effects of the early invitation, and the multiple micronutrient supplementation, on a range of cardiometabolic risk factors.

## Methods

### The MINIMat trial

The MINIMat trial (ISRCTN16581394) was conducted in Matlab sub-district, a rural area in Chandpur district, about 60 km south from Dhaka. The International Centre for Diarrhoeal Disease Research, Bangladesh (icddr,b) runs a Health and Demographic Surveillance System (HDSS) in a population of 220,000 in this area. As part of the HDSS activities, community health workers perform monthly household visits and collect demographic and health data. This surveillance was used to identify and enroll women as early as possible during pregnancy, over a 2-year period (2001–2003). The cohort of 3267 singleton children with a valid birth weight has subsequently been followed up. The original trial protocol is available at https://www.isrctn.com/ISRCTN16581394, and the effects on predefined outcomes (size and gestational age at birth, infant mortality and maternal haemoglobin and micronutrient status in third trimester) have been reported on earlier.^21^^,^^22^ The MINIMat cohort has then been followed up,^21^ and we now report on a follow-up at 15 years.

### Intervention

As described,^21^^,^^22^ the women had been individually and randomly allocated (2 by 3 factorial design) to two food groups and three micronutrient groups resulting in six groups in total (Fig. 1). The food supplement consisted of a powder made of roasted rice, roasted pulse, molasses and soybean oil, to be mixed with water, giving 608 kcal of energy and 18 g of protein per day. The two food supplement groups were invited to start supplementation either (i) immediately after detection of pregnancy, usually around pregnancy week 9 (early timing of invitation), or (ii) at the time of their choice, usually around pregnancy week 20 (usual timing of invitation, i.e. standard care). The three types of micronutrient supplements were capsules containing either: (i) 30 mg of iron (fumarate) and 400 μg folic acid (Fe30F); or (ii) 60 mg iron and 400 μg folic acid (Fe60F); or (iii) multiple micronutrients (MMS) containing 30 mg of iron, 400 μg of folic acid, 800 μg of RE vitamin A (retinyl acetate), 200 IU of vitamin D (D3), 10 mg of vitamin E (a-tocopherol acetate), 70 mg of vitamin C, 1.4 mg of vitamin B1 (thiamine mononitrate), 1.4 mg of vitamin B2 (riboflavin), 18 mg of niacin, 1.9 mg of vitamin B6 (pyridoxine hydrochloride), 2.6 μg of vitamin B12 (cyanocobalamin), 15 mg of zinc (sulphate), 2 mg of copper (sulphate), 65 μg of selenium (sodium selenite) and 150 μg of iodine (potassium iodide).

**Fig. 1:**
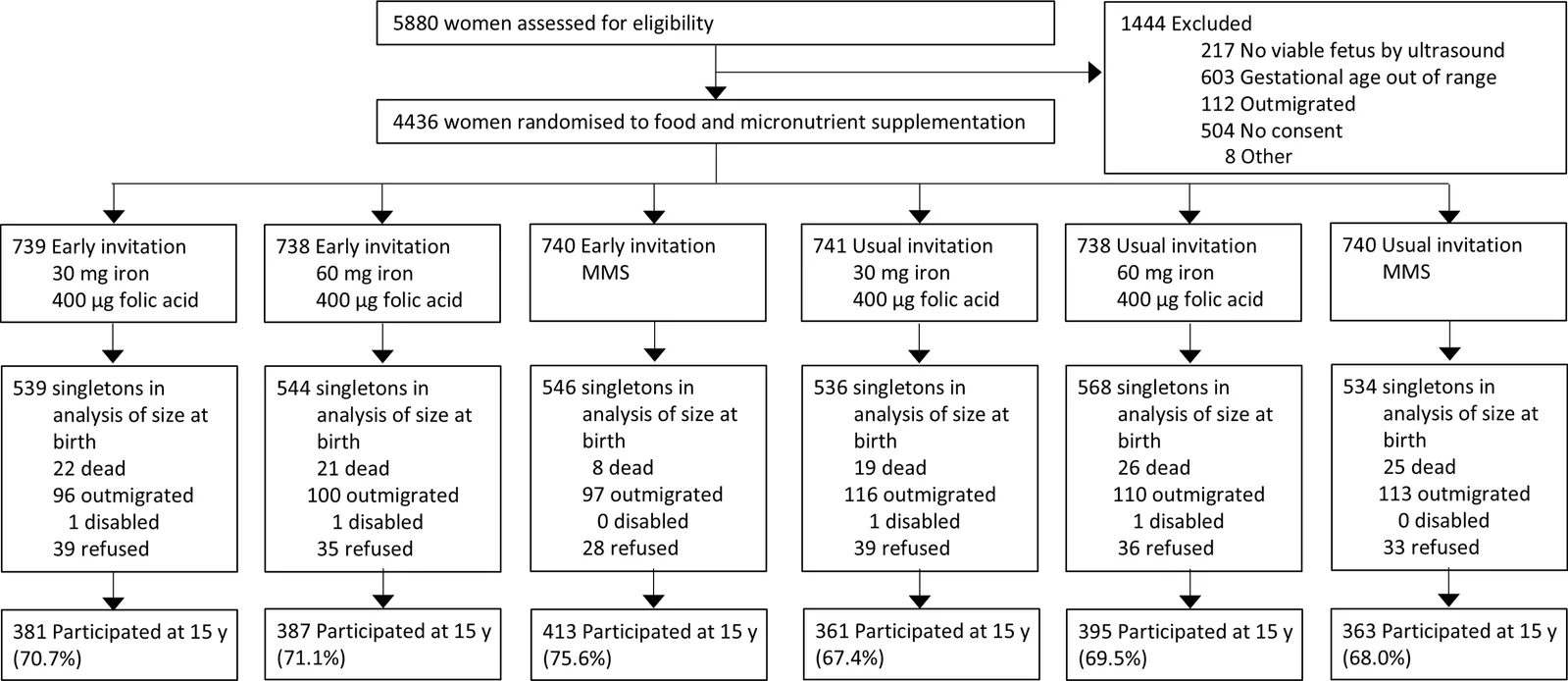
Flow chart of the study.

### 15-Year follow-up

Between August 2017 and June 2019, we invited the MINIMat cohort members for a clinical visit in the order of birth dates, close to the age of 15 y, to minimize difference due to age at the follow-up. Of the 3267 children in the cohort, 121 (3.7%) had died, 632 (19.3%) had out-migrated, 210 (6.4%) refused and 4 (0.1%) could not attend because of a severe disability (Fig. 1). All the 2300 (70.4%) participants had at least one cardiometabolic risk factor available (Table 1) and were included in the present study.

**Table 1:** Descriptive characteristics of mothers and adolescents by maternal food and micronutrient supplementation groups.

	N missing	All children	Maternal food supplementation		Maternal micronutrient supplementation		
			Early invitation	Usual invitation	MMS	Fe60F	Fe30F
		n = 2300	n = 1181	n = 1119	n = 776	n = 782	n = 742
**Maternal characteristics during pregnancy**							
Household SES (fifths)	0						
1 (Lowest)		459 (20.0%)	249 (21.1%)	210 (18.8%)	159 (20.5%)	152 (19.4%)	148 (19.9%)
2		496 (21.6%)	251 (21.3%)	245 (21.9%)	162 (20.9%)	170 (21.7%)	164 (22.1%)
3		480 (20.9%)	245 (20.7%)	235 (21.0%)	159 (20.5%)	159 (20.3%)	162 (21.8%)
4		462 (20.1%)	232 (19.6%)	230 (20.6%)	154 (19.8%)	169 (21.6%)	139 (18.7%)
5 (Highest)		403 (17.5%)	204 (17.3%)	199 (17.8%)	142 (18.3%)	132 (16.9%)	129 (17.4%)
Maternal age (y)	3	26.6 (6.0)	26.7 (6.1)	26.5 (5.8)	26.6 (6.0)	26.6 (6.1)	26.7 (5.9)
Parity (% primiparous)	0	676 (29.4%)	351 (29.7%)	325 (29.0%)	230 (29.6%)	224 (28.6%)	222 (29.9%)
Maternal height (cm)	3	149.8 (5.2)	149.8 (5.2)	149.7 (5.1)	149.8 (5.2)	149.8 (5.1)	149.7 (5.3)
Maternal early pregnancy BMI (kg/m^2^)	7	20.1 (2.6)	20.0 (2.6)	20.1 (2.6)	20.1 (2.5)	20.1 (2.7)	20.0 (2.5)
Maternal education (categories)	0						
No formal education		758 (33.0%)	399 (33.8%)	359 (32.1%)	255 (32.9%)	254 (32.5%)	249 (33.6%)
1–5 y		566 (24.6%)	280 (23.7%)	286 (25.6%)	178 (22.9%)	189 (24.2%)	199 (26.8%)
6 y or more		976 (42.4%)	502 (42.5%)	474 (42.4%)	343 (44.2%)	339 (43.4%)	294 (39.6%)
**Newborn characteristics**							
Boys	0	1101 (47.9%)	563 (47.7%)	538 (48.1%)	377 (48.6%)	376 (48.1%)	348 (46.9%)
Length of gestation (weeks)	182	39.3 (1.8)	39.3 (1.8)	39.2 (1.8)	39.3 (1.8)	39.3 (1.8)	39.2 (1.9)
Birth weight (g)	1	2691 (393)	2699 (389)	2682 (397)	2690 (384)	2696 (378)	2686 (417)
Birth weight z score^a^	207	−1.25 (0.92)	−1.23 (0.90)	−1.27 (0.93)	−1.27 (0.91)	−1.26 (0.91)	−1.22 (0.92)
**Current maternal and household characteristics**							
Maternal education	0						
No formal education		457 (19.9%)	245 (20.7%)	212 (18.9%)	141 (18.2%)	159 (20.3%)	157 (21.2%)
1–5 y		812 (35.3%)	407 (34.5%)	405 (36.2%)	276 (35.6%)	264 (33.8%)	272 (36.5%)
6 y or more		1031 (44.8%)	530 (44.8%)	502 (44.9%)	359 (46.3%)	359 (45.9%)	313 (42.2%)
Source of drinking water	0						
Piped water		138 (6.0%)	65 (5.5%)	73 (6.5%)	54 (7.0%)	48 (6.1%)	36 (4.9%)
Tube-well water		2130 (92.6%)	1100 (93.5%)	1030 (92.0%)	709 (90.7%)	722 (92.3%)	701 (94.5%)
Rain, river or unknown		32 (1.4%)	16 (1.4%)	16 (1.4%)	13 (1.7%)	12 (1.5%)	5 (0.7%)
Latrine toilet	10	441 (19.2%)	225 (19.1%)	216 (19.4%)	153 (19.8%)	145 (18.7%)	143 (19.3%)
Electricity	0	2033 (88.4%)	1043 (88.3%)	990 (88.5%)	699 (90.1%)	686 (87.7%)	648 (87.3%)
**Adolescent characteristics**							
Participant education	0						
No formal education		364 (15.8%)	182 (15.4%)	182 (16.3%)	130 (16.8%)	124 (15.9%)	110 (14.8%)
1–5 y		58 (2.5%)	26 (2.2%)	32 (2.9%)	20 (2.6%)	20 (2.6%)	18 (2.4%)
6 y or more		1878 (81.7%)	973 (82.4%)	905 (80.9%)	626 (80.7%)	638 (81.6%)	614 (82.7%)
Age (y)	0	15.0 (0.1)	15.0 (0.1)	15.0 (0.1)	15.0 (0.2)	15.0 (0.2)	15.0 (0.1)
Height (cm) girls	2	152.9 (5.4)	153.0 (5.2)	152.8 (5.5)	152.9 (5.4)	152.9 (5.4)	152.7 (5.4)
Height (cm) boys	2	160.4 (7.7)	161.0 (7.2)	159.9 (8.3)	160.2 (7.7)	160.8 (7.7)	160.3 (7.7)
BMI (kg/m^2^) girls	2	19.0 (3.2)	18.9 (3.3)	19.1 (3.2)	19.1 (3.2)	19.1 (3.3)	18.9 (3.3)
BMI (kg/m^2^) boys	2	17.8 (2.8)	17.9 (2.9)	17.6 (2.7)	17.7 (2.7)	18.0 (3.1)	17.6 (2.6)
Height z score	2	−1.24 (0.89)	−1.20 (0.84)	−1.28 (0.94)	−1.23 (0.89)	−1.22 (0.89)	−1.26 (0.89)
Stunted, n (%)	2	445 (19.4%)	205 (17.4%)	240 (21.4%)	154 (19.9%)	140 (17.9%)	151 (20.4%)
BMI z score	2	−0.94 (1.26)	−0.93 (1.26)	−0.95 (1.26)	−0.94 (1.26)	−0.90 (1.28)	−1.00 (1.25)

The numbers are n (%) or mean (SD).

Fe30F, 30 mg of iron and 400 μg folic acid; Fe60F, 60 mg of iron and 400 μg folic acid; MMS, multiple micronutrient supplementation.

a Less than 10th percentile for gestational age by INTERGROWTH birth weight reference.

### Outcomes

Height was measured by Leicester Height Measure stadiometer (Seca Ltd, Birmingham, UK) and weight by Tanita TBF-300 MA BC-418 Body Composition Analyzer (Tanita Corporation, Tokyo, Japan) and transferred to z scores using the WHO reference,^28^ BMI was calculated as kg/m^2^.

As described in detail,^29^ blood samples were obtained after an overnight fast to a lithium-heparin tube, transported within 2 h on ice to the Matlab laboratory, with plasma separated and frozen in −70 C. high-density lipoprotein (HDL), LDL, total cholesterol, triglycerides, creatinine and cystatin C were analysed by a colorimetric test and apolipoprotein A1 and B by immunoturbidimetry using the Cobas® automatic analyser at the laboratory of the Department of Clinical Chemistry, Skåne University Hospital, Sweden. The laboratory is accredited for these analyses.

Insulin concentrations were measured in icddr,b laboratory, Bangladesh, by an automated immunoassay analyzer, Cobas e601 (Roche Diagnostics GmbH, Mannheim, Germany) based on electrochemiluminescence immunoassay (ECLIA) using commercial kits according to manufacturer's instruction. This method has been standardized using the 1st International Reference Preparation (IRP) WHO Reference Standard 66/304 (National Institute for Biological Standards and Control NIBSC). Results were determined via a calibration curve which was instrument specifically generated by 2-point calibration and a master curve provided via the reagent barcode. Two level quality control serum, PreciControl Multimarker (Roche Diagnostics, GmbH, D-68298 Mannheim, Germany) were used as an internal quality control for monitoring both the accuracy and precision of this assay.

Concentrations of CRP and sex-hormone binding globulin (SHBG) were measured using a highly sensitive electrochemiluminescence immunoassay—the R-PLEX Human CRP and SHBG Assay kit form Meso-Scale Discovery (MSD) according to the manufacturer's instructions–at the Institute of Environmental Medicine, Stockholm, Sweden. The intra and inter-coefficient of variation for an in-house plasma sample was 2.9 and 4.4% for CRP and 4.9 and 5.3% for SHBG. Glucose was measured using the Contour TS Blood Glucose Monitoring System (Bayer) during the clinic visit.

As a composite cardiometabolic score, we used a sum of z scores for waist circumference, HDL cholesterol (inverted), triglycerides, fasting blood glucose and mean of systolic and diastolic blood pressure.^30^ As a measure of insulin resistance we used Homeostasis Model Assessment equation index (HOMA-IR), calculated as (fasting insulin mU/L × fasting glucose in mmol/L) ÷ 22.5.

We measured blood pressure by using an automated oscillometric device (Omron HEM-7120, Morton Medical). We recorded the measurements in triplicate. The first measurement was taken after the participant had been seated at rest for a minimum of 10 min, and the subsequent measurements were 2 min apart. The arithmetic mean of the second and third measurements was used in analysis.

### Data analysis and covariates

We used multiple linear regression to assess the effects of the intervention on continuous outcomes (height and BMI z sores, cardiometabolic biomarkers, blood pressure and a composite cardiometabolic risk score) and logistic regression on a dichotomous outcome (stunting). As in previous publications,^12^^,^^25^^,^^26^ we conducted two comparisons: early invitation with usual as reference, and multiple micronutrients with 400 μg folic acid and 60 mg iron, the routine supplementation at that time, as reference. There was no interaction between the effects of early invitation and multiple micronutrients on any of the outcomes. We present an unadjusted model and a model adjusting for participant sex and age and potential pregnancy confounders: maternal age, maternal height and asset score originally generated through principal component analysis of household assets which were further grouped by quintiles.^31^ In an additional model, we also adjust for maternal primiparity, education and BMI in early pregnancy, plus participant current BMI. Because of very small numbers of missing values for covariates (maximum 7, for maternal BMI), we conduct complete case analyses. Upon routine inspection of the biochemical measurement data we found that there was a correlation (r = −0.20) between the sample storage time and log-transformed insulin concentration. We therefore adjust for sample storage time in all analyses including insulin concentrations. We tested for interactions with sex by adding a product term intervention∗sex in the regression equation.

We log-transformed biochemical analysis data to attain normality, back-transformed their regression coefficients and express them as percent difference between the exposure variable categories. Current characteristics in Table 1, collected by structured interview, were not used in analyses but are presented to describe the study setting.

### Ethics

The reported study was nested into the 15-year follow-up of the MINIMat trial, which has been approved by the Ethical Review Committee at icddr,b in Dhaka, Bangladesh (PR-17029; date 2017/05/23). We obtained written informed consent from the mothers and assent from the adolescents. Patients or the public were not involved in the design, or conduct, or reporting, or dissemination plans of our research.

### Role of the funding sources

The funders had no role in study design, data collection, data analysis, interpretation or writing of the report.

## Results

### Participants and nonparticipants

Characteristics of the 2300 participants of the 15-year follow-up study, compared with the remaining 967 members of the MINIMat Follow-up cohort (Fig. 1), are shown in Supplementary Table S1, which also shows these comparisons within each food or micronutrient supplementation group. Adolescents whose mothers had been randomised to the early invitation group were more likely to participate at 15 years (OR 1.22 compared with usual invitation; 95% CI 1.05, 1.42), but participation was unrelated to multiple micronutrient supplementation (OR 1.08 compared with 60 mg iron and folic acid; 95% CI 0.90, 1.30). Girls had a higher participation rate than boys. Higher participation rate was also associated with lower maternal SES or education, multiparity and lower pre-pregnancy BMI.

### Participant characteristics

Characteristics of the 2300 participants (1199 boys; 47.9%) are shown in Table 1. During pregnancy their mothers were short and lean, with a mean height of 149.8 cm and BMI of 20.1 kg/m^2^. A third of them had no formal education. The babies were born on average 39.2 weeks of gestation with a mean birth weight of 2691 g. Of them, 9.0% were born preterm (<37 weeks), 31.7% had low birth weight (<2500 g) and 46.2% were born small for gestational age (<10th percentile). At 15 years of age, over 98% of the adolescents had access to tube-well or piped water; 19% had a latrine toilet and over 88% had electricity in the household. While over 80% had attended more than primary school (6 years or more of education), 16% had no formal education.

### Cardiometabolic and anthropometric outcomes at 15 y by prenatal food supplementation

Concentrations of cardiometabolic markers in the study population and by intervention group are shown in Table 2 and their association with height and BMI z scores in Supplementary Table S2. There were no differences between early and usual invitation groups, or between MMS and 60 mg iron and folic acid groups (Table 3). Children in the early invitation group had a 0.08 units (95% CI 0.01, 0.16) higher height z score than those in the usual invitation group, corresponding to 0.6 cm (0.0, 1.2). They were also less likely to be stunted (17.4% vs 21.4%, absolute risk difference 4.1%, 95% CI 0.8%, 7.3%, OR 0.77; 95% CI 0.63, 0.95). There was no difference in BMI z score (Table 3). Adjustment for pregnancy-related covariates and current participant BMI had a negligible effect on the results (Table 3, Supplementary Table S3).

**Table 2:** Cardiometabolic markers by maternal food and micronutrient supplementation groups.

	All children	Early invitation	Usual invitation	MMS	Fe60F	Fe30F	n missing
	n = 2300	n = 1181	n = 1119	n = 776	n = 782	n = 742	
Total cholesterol (mmol/l)	3.51 (1.20)	3.51 (1.20)	3.50 (1.20)	3.49 (1.21)	3.50 (1.20)	3.52 (1.20)	55
LDL cholesterol (mmol/l)	2.06 (1.30)	2.05 (1.30)	2.07 (1.30)	2.06 (1.30)	2.06 (1.30)	2.06 (1.30)	55
HDL cholesterol (mmol/l)	0.99 (1.24)	1.00 (1.23)	0.99 (1.25)	0.99 (1.23)	1.01 (1.25)	0.99 (1.24)	55
Triglycerides (mmol/l)	0.95 (1.51)	0.96 (1.51)	0.94 (1.50)	0.94 (1.49)	0.93 (1.49)	0.98 (1.54)	55
Apolipoprotein A1 (g/l)	1.03 (1.16)	1.04 (1.16)	1.03 (1.16)	1.03 (1.15)	1.04 (1.17)	1.04 (1.16)	55
Apolipoprotein B (g/l)	0.70 (1.24)	0.70 (1.24)	0.70 (1.24)	0.70 (1.25)	0.70 (1.24)	0.70 (1.24)	57
Glucose (mmol/l)	5.17 (1.09)	5.19 (1.10)	5.15 (1.09)	5.17 (1.09)	5.17 (1.10)	5.16 (1.10)	47
Insulin (pmol/l)	12.7 (2.09)	13.0 (2.07)	12.5 (2.10)	12.7 (2.07)	12.8 (2.07)	12.7 (2.12)	55
HOMA-IR	0.24 (2.10)	0.25 (2.08)	0.24 (2.11)	0.24 (2.08)	0.24 (2.08)	0.24 (2.13)	55
Sex hormone binding globulin (nmol/l)	37.0 (1.70)	36.9 (1.69)	37.2 (1.70)	36.7 (1.70)	36.6 (1.69)	37.8 (1.69)	51
Creatinine (μmol/l)	50.8 (1.21)	50.6 (1.21)	51.0 (1.21)	50.8 (1.21)	51.1 (1.20)	51.1 (1.21)	55
Cystatin C (mg/l)	0.87 (1.18)	0.86 (1.18)	0.86 (1.18)	0.87 (1.18)	0.87 (1.18)	0.86 (1.18)	55
CRP (mg/l)	0.47 (3.97)	0.45 (3.86)	0.50 (4.08)	0.48 (3.97)	0.47 (4.09)	0.47 (3.88)	51
Systolic blood pressure (mmHg)^a^	108.8 (8.0)	109.0 (7.8)	108.6 (8.2)	108.5 (8.3)	109.1 (8.0)	108.8 (7.7)	1
Diastolic blood pressure (mmHg)^a^	62.9 (7.5)	63.1 (7.4)	62.8 (7.6)	62.8 (7.7)	63.2 (7.5)	62.7 (7.2)	1
Cardiometabolic risk score^a^	−0.00 (0.61)	0.02 (0.63)	−0.02 (0.60)	−0.01 (0.60)	−0.01 (0.61)	0.02 (0.63)	61

The numbers are geometric means (SDs). The geometric mean is the nth root of the product of n individual values. Geometric SDs correspond to the percentage increase in a variable corresponding to one SD unit change in the logarithm of the variable.

Fe30F, 30 mg of iron and 400 μg folic acid; Fe60F, 60 mg of iron and 400 μg folic acid; HDL, high-density lipoprotein; HOMA-IR, Homeostasis model assessment for insulin resistance equation: (fasting insulin in mU/L × fasting glucose in mmol/L) ÷ 22.5; LDL, low-density lipoprotein; MMS, multiple micronutrient supplementation.

a Arithmetic means and SDs.

**Table 3:** Mean differences in body size, cardiometabolic markers and blood pressure, and odds ratios for stunting according to early compared with usual invitation to food supplementation, and according to multiple micronutrient supplementation (MMS) compared with 60 mg of iron and 400 μg folic acid (Fe60F).

	Early invitation compared with usual invitation, crude mean difference (95% CI)	Early invitation compared with usual invitation, adjusted^a^ mean difference (95% CI)	MMS compared with Fe60F, crude mean difference (95% CI)	MMS compared with Fe60F, adjusted^a^ mean difference (95% CI)
Height z score	0.08 (0.01, 0.16)	0.09 (0.02, 0.16)	−0.01 (−0.10, 0.07)	−0.01 (−0.10, 0.07)
Stunted (odds ratio)	0.77 (0.63, 0.95)	0.74 (0.60, 0.92)	1.14 (0.98, 1.45)	1.13 (0.87, 1.47)
BMI z score	0.02 (−0.08, 0.13)	0.04 (−0.06, 0.14)	−0.02 (−0.15, 0.10)	−0.02 (−0.14, 0.10)
Total cholesterol (mmol/l)	0.2% (−1.3%, 1.7%)	0.2% (−1.3%, 1.7%)	−0.3% (−2.1%, 1.6%)	−0.3% (−2.1%, 1.5%)
LDL cholesterol (mmol/l)	−0.8% (−2.9%, 1.4%)	−0.7% (−2.8%, 1.5%)	−0.3% (−2.9%, 2.4%)	−0.3% (−2.9%, 2.3%)
HDL cholesterol (mmol/l)	0.9% (−0.9%, 2.7%)	0.9% (−0.9%, 2.7%)	−2.0% (−4.1%, 0.2%)	−2.0% (−4.1%, 0.1%)
Triglycerides (mmol/l)	1.8% (−1.6%, 5.3%)	1.5% (−1.8%, 5.0%)	1.4% (−2.6%, 5.5%)	1.4% (−2.5%, 5.5%)
Apolipoprotein A1 (g/l)	0.7% (−0.6%, 2.0%)	0.7% (−0.6%, 2.0%)	−1.0% (−2.5%, 0.5%)	−1.0% (−2.5%, 0.5%)
Apolipoprotein B (g/l)	−0.8% (−2.6%, 1.0%)	−0.7% (−2.4%, 1.0%)	0.2% (−2.0%, 2.4%)	0.2% (−1.9%, 2.3%)
Glucose (mmol/l)	0.7% (−0.1%, 1.4%)	0.6% (−0.1%, 1.4%)	−0.1% (−1.0%, 0.8%)	−0.1% (−1.0%, 0.8%)
Insulin (pmol/l)^b^	4.2% (−1.8%, 10.6%)	4.3% (−1.7%, 10.6%)	−0.2% (−7.1%, 7.2%)	−0.2% (−7.0%, 7.0%)
HOMA-IR^b^	4.4% (−1.7%, 10.8%)	4.4% (−1.6%, 10.7%)	−0.2% (−7.2%, 7.2%)	−0.2% (−7.0%, 7.1%)
Sex hormone binding globulin (nmol/l)	−0.6% (−4.9%, 3.8%)	−1.6% (−5.5%, 2.5%)	0.3% (−4.9%, 5.7%)	0.2% (−4.7%, 5.3%)
Kreatnine (μmol/l)	−0.7% (−2.3%, 0.9%)	−0.5% (−1.8%, 0.9%)	−0.6% (−2.5%, 1.3%)	−0.6% (−2.2%, 1.1%)
Cystatin C (mg/l)	0.3% (−1.1%, 1.7%)	0.3% (−0.7%, 1.4%)	0.1% (−1.6%, 1.8%)	0.0% (−1.2%, 1.3%)
CRP (mg/l)	−9.6% (−19.4%. 1.3%)	−8.3% (−18.2%, 2.7%)	1.2% (−12.0%, 16.4%)	1.8% (−11.5%, 17.1%)
Systolic blood pressure (mmHg)	0.4 (−0.2, 1.1)	0.4 (−0.2, 1.2)	−0.5 (−1.3, 0.3)	−0.6 (−1.3, 0.2)
Diastolic blood pressure (mmHg)	0.3 (−0.3, 0.9)	0.3 (−0.3, 0.9)	−0.4 (−1.1, 0.4)	−0.4 (−1.1, 0.4)
Cardiometabolic risk score	0.035 (−0.016, 0.086)	0.037 (−0.014, 0.088)	0.005 (−0.056, 0.066)	0.006 (−0.055, 0.067)

Additional adjustments are shown in Supplementary Table S3.

a Adjusted for sex, age and maternal variables during pregnancy: asset score, maternal height and maternal age.

b Adjusted in addition for sample storage time.

Mean total cholesterol concentrations were 3.51 mmol/l and LDL cholesterol concentrations 2.06 mmol/l, and 135 participants (6.0%) had an LDL cholesterol concentration of 3.0 mmol/l or more and 12 (0.5%) over 4.0 mmol/l. These proportions were similar between groups. Mean systolic and diastolic blood pressures were 108.8 mmHg and 62.9 mmHg, and 182 (7.9%) participants had a blood pressure exceeding 120 or 80 mmHg and 13 participants (0.6%) exceeding 130 mmHg or 85 mmHg. Again, the proportions were similar between groups.

Of the participants, 67 (3.0%) had a CRP value greater than 10 mg/l, which may indicate acute infection. Of them, 27 were in the early and 40 in usual invitation group (χ^2^ p = 0.07); 18 were in the 30Fe, 24 in 60Fe and 25 in MMS group (p = 0.6). After exclusion of participants with CRP greater than 10 mg/l (Table 3), the results were little changed (crude mean difference with food type −4.6%; −14.1%, 5.8%; with micronutrient supplementation 0.7%; −11.2%, 14.3%).

### Sex interactions

We then tested whether the associations between the intervention and the outcomes were different in female and male participants. With two exceptions, there was no interaction between the effects of sex and either food or multiple micronutrient supplementation on the outcomes in Table 3. We therefore present the results with both sexes pooled, adjusting for sex and other covariates. One of the two exceptions was an interaction between the effects of sex and early invitation to supplementation on apolipoprotein A1 (p = 0.03). Among females, early invitation was associated with 2.1% (0.3%, 3.9%) higher apolipoprotein A1 and 2.6% (0.0%, 5.2%) higher HDL cholesterol concentrations. This was not seen among males (corresponding differences were −0.7%; −2.5% to 1.1% and −0.8%; −3.4% to 1.8%). Another interaction (p = 0.03) was seen between sex and MMS on CRP; however, in sex-stratified analyses there was no statistically significant association between MMS and CRP in either sex.

## Discussion

In this follow-up of a pregnancy randomised controlled trial, our primary hypothesis was that the effects of early food supplementation on taller height and lower LDL cholesterol and apolipoprotein B concentrations, observed at 4.5 y, would be sustained at 15 y. This hypothesis was true for height, although the magnitude was small, 0.08 unit difference in z height z score, which was similar to that in the follow-up at 4.5 y. However, it was parallelled by a reduction of stunting from 21.4% to 17.4%, the risk difference of 4.1% corresponding to a number needed to treat of 24. Height was unrelated to maternal multiple micronutrient supplementation. We found no effect of the interventions on adolescent offspring LDL cholesterol or apolipoprotein B concentrations or on BMI, markers of glucose metabolism, kidney function, inflammation or blood pressure.

A few maternal protein-energy supplementation interventions in undernourished populations have assessed anthropometry and cardiometabolic risk factors in the child or adult offspring.^6^ In India, the Hyderabad nutrition trial cluster-randomised mothers to receive a protein (20 g) and energy (2.51 MJ) supplementation, and continued with a half dose to the child up to 6 y. Similar to MINIMat, fertile age women were visited once a month to ensure early detection of pregnancy and early randomization to supplementation or usual care.^9^ At age 13–18 y, the adolescents were 1.0 cm taller, had lower measures of insulin resistance and arterial stiffness.^9^ In a trial in The Gambia, mothers received protein (22 g) and energy (1015 kcal) from 20 weeks to delivery. This intervention increased birth weight by 136 g.^32^ At age 11 to 17 y, the intervention children were marginally shorter^13^ and had lower fasting glucose^10^ but there were no differences in plasma cholesterol,^10^ blood pressure^33^ or body composition.^13^ The INCAP study in Guatemala cluster-randomised mothers and children by village to receive daily either a high-energy or high-protein drink or lower-energy drink, consumed in variable quantities. As young adults 450 of them were assessed. At that time, the prenatal supplementation was associated with higher HDL cholesterol and lower triglyceride concentrations,^11^ lower plasma glucose among women only^8^ but not with blood pressure,^34^ However, the INCAP prenatal supplementation was also associated with an implausibly large effect on height (12 cm among women and 13.5 cm among men),^34^ warranting caution for the other outcomes as well. Taken together, the two trials, MINIMat and Hyderabad maternal nutrition trial, which aimed at pregnancy identification and supplementation as early as possible, show increased offspring height, which is not seen in The Gambia trial with supplementation from week 20 onwards. This argues for the importance of adequate protein and energy intake during first and early second trimesters in terms of offspring body size. While some of these studies suggest marginal benefit on glucose metabolism, this effect was not seen in the present study, and overall there is little evidence benefits of maternal protein-energy supplementation on cardiometabolic risk factors in adolescence. Levels of risk factors and percentage of participants with abnormal levels were low, and we cannot exclude effects that might arise later in life when risk factor levels increase.

Interaction analyses showed that the effects of early invitation to food supplementation on adolescent body size and cardiometabolic risk factors, or the lack of these effects, are similar in girls and boys. An exception was the effects of early invitation on higher adolescent concentrations of HDL cholesterol and its main component apolipoprotein A1 seen among girls but not among boys. To our knowledge similar effects of early food supplementation have not been reported before. However, because of the large number of comparisons, we cannot exclude that this may be a chance finding. Therefore, unless replicated, we would like to interpret it with caution.

A number of randomised trials have assessed offspring effects of maternal multiple micronutrient supplementation. Most focus on the first two years of life: a recent meta-analysis of 19 trials concluded that MMS, compared with iron and folic acid, result in greater body size from birth to 6 months but not thereafter.^35^ As to studies with longer follow-up, a multiple micronutrient trial in pregnant women in Nepal that had resulted in 77 g increase in birth weight showed no difference in height but increased weight, chest, hip and middle upper-arm circumferences, accompanied by lower systolic blood pressure in 2.5-year-old children than children whose mothers received standard iron/folate tablets.^14^ The effects on body size were reassessed at 8.5 years, but none of them were present at that point.^20^ Another trial in Nepal compared multiple micronutrients with no supplementation and included in addition groups receiving folic acid, folic acid + iron and folic acid + iron + zinc. At age 6–8 years, there was no difference in body size,^17^ body composition assessed by skinfolds^17^ or metabolic syndrome or plasma cardiometabolic biomarkers^16^ between multiple micronutrient and control groups. The Mumbai Maternal Nutrition Project randomised fertile-aged women to receive micronutrient-rich or control foods starting preconceptionally. Among children of women who received the supplement more than 3 months before pregnancy, the intervention resulted in 48 g higher birth weight.^36^ At age 5–10 y, the children were similar in terms of height, plasma lipids levels, blood pressure and insulin resistance, but girls in the intervention group had higher BMI and body fat percentage.^18^ A study in 10-14-year-olds in rural Shaanxi, China compared multiple micronutrients with folic acid only and found lower blood pressure.^15^ Together with our findings, no consistent picture seems to have emerged. Any effect of maternal MMS on offspring body size and cardiometabolic risk factors is likely to be context specific.

Of other types of nutritional intervention, small quantity lipid-nutrient supplements improve linear growth according to a meta-analysis up to 2 y of age,^37^ although individual studies with longer follow-up may not show a sustained effect.^38^ Nutrition is also a key component in multidomain interventions including the Wings study in India^39^ and the ongoing HeLTI^40^ interventions in India, China, South Africa and Canada, aiming to reduce childhood adiposity.

A difference of 0.08 SD, or 0.6 cm, in height caused by early invitation to food supplementation, was accompanied by a reduction in stunting rates from 21.4% to 17.4%, with a risk difference of 4.1% corresponding to 24 mothers needed to be treated to prevent one case of stunting at 15 y. This is similar to 4.5 y, when the analogous reduction of stunting rate was 35.3% to 30.8%,^23^ the risk difference of 4.5% corresponding to a number needed to treat of 22. The cost of the MINIMat early food intervention at 2015 cost levels has been estimated at 79 to 86 USD per mother (depending on micronutrient regime).^41^^,^^42^ Based on data at 4.5 y, averting one disability-adjusted life year due to death, developmental delay or stunting has been estimated to cost 24 USD.^41^ While the effect on stunting alone may be too small to prompt changes in policies, the finding serves as proof or principle that, consistent with the DOHaD theory, the effects of food supplementation early in pregnancy on body size persist at least to adolescence.

Strengths of the study include the very well phenotyped MINIMat cohort with adequate power to confirm or exclude clinically meaningful differences between intervention groups. Possibility of selection bias is a limitation characteristic for long-term follow-up studies. Participation rate, 70.4% of the original child cohort and 73.2% of those alive and not disabled, compares favourably with corresponding pregnancy interventions followed up in adolescence: 64.3% of liveborns at mean 14 years in The Gambia,^10^ 44.8% at 15 y in the Hyderabad Nutrition Trial^9^ and 44.4% at 14 years in a trial in China.^15^ Somewhat higher follow-up rates have been reported for younger children: micronutrient trials in Nepal report 93% at 6–8 y^16^ and 80% in another trial at 8.5 y,^20^ whereas the Mumbai Maternal Nutrition trial reports 66% at 5–10 years.^18^ Participation rates were higher among girls and among those whose mothers had lower socio-economic position; adjustment for these factors had no effect on the associations. Of note, adolescents whose mothers received early invitation to food supplementation were slightly more likely to participate in the 15-year assessment than their counterparts in the usual invitation group. While this invites speculation on potential health benefits of early food supplementation, this would only be expected to introduce bias if the association between early intervention and the outcomes would be different among participants and nonparticipants. This cannot be excluded and is a limitation of the study. A further limitation is that pubertal status was not assessed at 15-year visit. In a previous MINIMat puberty assessment at 12–15 years, girls and boys with a history of stunting had attained pubertal changes, growth spurt and menarche later than others.^43^ It remains possible that the taller height and lower risk of stunting we found in the early food supplementation group in part indicate earlier puberty. However, food or micronutrient supplementation was not associated with age at menarche in MINIMat,^44^ making this an unlikely mediator at least among girls. Association of food or micronutrient supplementation with boys’ timing of puberty was not reported.^44^

To conclude, in a population where undernutrition is prevalent, the effect of early invitation to food supplementation on greater height of offspring and reduced rates of stunting is sustained at 15 years. While this difference is modest, it serves as a proof of principle of an effect of early pregnancy nutrition intervention persisting in the offspring at least up to adolescence, in accordance with the DOHaD theory. We were unable to see any consistent effects on lipid profile, other cardiometabolic biomarkers or blood pressure.

## Contributors

EK and ECE had directly accessed and verified the underlying data that used in this manuscript. EK, RR, ECE, PNG, SMR, MK and AR conceptualised and designed the study. EK, RR, ECE, MR, PNG, SMR, JP, MK and AR oversaw data collection. EK, ECE, MR, SMR, JP, MRI and MK performed data curation. EK designed and carried out the analysis. EK, ECE, SMR, SEA, MK and AR acquired the funding for this study. EK drafted the initial manuscript and coordinated other authors’ contributions. RR, ECE, MR, PNG, SMR, JP, MRI, SEA, ML and AR participated in review and editing of the final manuscript. All authors have access to all the data reported in the study and have read and approved the submission of the final version of the manuscript.

## Data sharing statement

The present study utilized data from the 15-year follow-up of the MINIMat (Maternal and Infant Nutrition Interventions in Matlab) trial. The 15-year follow-up was a large, collaborative project involving International Centre for Diarrhoeal Disease Research, Bangladesh (icddr,b), Uppsala University, Karolinska Institute, Finnish Institute for Health and Welfare and University of Oulu. Because of the statutory requirements, internal data policies and regulations existing in the collaborating bodies along with the over-arching European Union General Data Protection Regulation (GDPR), the data must be stored in institutional repository and cannot be made directly accessible without a review of the request for access to data. Data availability is further limited because the data contain sensitive health information. Therefore, the data can be accessed only upon a formal request that details the purpose of such request. Requests for data access should be directed to: E-CE (email: lotta. ekstrom@kbh.uu.se).

## Declaration of interests

EK has been supported by Research Council of Finland, Finska Läkaresällskapet, Foundation for Pediatric Research, Novo Nordisk Foundation, Signe and Ane Gyllenberg Foundation and Sigrid Jusélius Foundation for the present manuscript and in addition European Commission for other work. SEA Has been supported by Swedish Research Council for the present manuscript. Other authors report no conflict of interest.
