# Supplementary material for: Effects of prenatal early food and micronutrient supplementations on body size and cardiometabolic risk factors in the 15-year-old offspring – the MINIMat randomised trial
Source: Lancet Reg Health Southeast Asia. 2026 Oct 5;55:100878. doi: 10.1016/j.lansea.2026.100878 (PMC13672831; doi:10.1016/j.lansea.2026.100878)
Supplement: Supplementary file 1 [file mmc1.docx]

Supplementary table 1. Non-participant analysis.

|  |  | | **Maternal food supplementation** | | | | **Maternal micronutrient supplementation** | | | | | |
| --- | --- | --- | --- | --- | --- | --- | --- | --- | --- | --- | --- | --- |
|  | **All children** | | **Early invitation** | | **Usual invitation** | | **MMS** | | **Fe60F** | | **Fe30F** | |
|  | Participated | | Participated | | Participated | | Participated | | Participated | | Participated | |
|  | Yes | No | Yes | No | Yes | No | Yes | No | Yes | No | Yes | No |
| Total number | 2300 (70.4%) | 967 (29.6%) | 1181 (72.5%) | 448 (27.5%) | 1119 (68.3%) | 519 (31.7%) | 776 (71.9%) | 304 (28.1%) | 782 (70.3%) | 330 (29.7%) | 742 (69.0%) | 333 (31.0%) |
| Household SES (fifths) | † |  |  |  | § |  |  |  | † |  |  |  |
| 1 (Lowest) | 459 (69.9%) | 198 (30.1%) | 249 (72.2%) | 96 (27.8%) | 210 (67.3%) | 102 (32.7%) | 159 (73.6%) | 57 (26.4%) | 152 (70.0%) | 65 (30.0%) | 148 (66.1%) | 76 (33.9%) |
| 2 | 496 (73.7%) | 177 (26.3%) | 251 (76.8%) | 76 (23.2%) | 245 (70.8%) | 101 (29.2%) | 162 (73.0%) | 60 (27.0%) | 170 (73.9%) | 60 (26.1%) | 164 (74.2%) | 57 (25.8%) |
| 3 | 480 (73.8%) | 170 (26.2%) | 245 (74.0%) | 86 (26.0%) | 235 (73.7%) | 84 (26.3%) | 159 (75.7%) | 51 (24.3%) | 159 (73.3%) | 58 (26.7%) | 162 (72.6%) | 61 (27.4%) |
| 4 | 462 (71.2%) | 187 (28.8%) | 232 (71.6%) | 92 (28.4%) | 230 (70.8%) | 95 (29.2%) | 154 (71.6%) | 61 (28.4%) | 169 (75.1%) | 56 (24.9%) | 139 (66.5%) | 70 (33.5%) |
| 5 (Highest) | 403 (63.2%) | 235 (36.8%) | 204 (67.5%) | 98 (32.5%) | 199 (59.2%) | 137 (40.8%) | 142 (65.4%) | 75 (34.6%) | 132 (59.2%) | 91 (40.8%) | 129 (65.2%) | 69 (34.8%) |
| Maternal age (y) | 26.6* (6.0) | 26.1 (5.7) | 26.7 (6.1) | 26.3 (5.7) | 26.5 (5.8) | 25.9 (5.8) | 26.6 (6.0) | 26.6 (5.9) | 26.6 (6.1) | 26.0 (5.7) | 26.7* (5.9) | 25.8 (5.6) |
| Primiparous | 676§ (65.7%) | 353 (34.3%) | 351* (68.6%) | 161 (31.4%) | 325† (62.9%) | 192 (37.1%) | 230 (68.0%) | 108 (32.0%) | §224 (63.6%) | 128 (36.4%) | 222 (65.5%) | 117 (34.5%) |
| Multiparous | 1624§ (72.6%) | 614 (27.4%) | 830* (74.3%) | 287 (25.7%) | 794† (70.8%) | 327 (29.2%) | 546 (73.6%) | 196 (26.4%) | §558 (73.4%) | 202 (26.6%) | 520 (70.7%) | 216 (29.3%) |
| Maternal height (cm) | 149.8 (5.2) | 149.9 (5.5) | 149.8 (5.2) | 150.1 (5.3) | 149.7 (5.1) | 149.8 (5.6) | 149.8 (5,2) | 149.9 (5.6) | 149.8 (5.1) | 150.1 (5.4) | 149.7 (5.3) | 149.8 (5.5) |
| Maternal early-pregnancy BMI (kg/m2) | 20.1* (2.6) | 20.3 (2.8) | 20.0* (2.6) | 20.3 (2.7) | 20.1 (2.6) | 20.3 (2.8) | 20.1§ (2.5) | 20.7 (2.9) | 20.1 (2.7) | 20.2 (2.6) | 20.0 (2.5) | 20.1 (2.8) |
| Maternal education | § |  | † |  | § |  | * |  | § |  | † |  |
| No | 758 (71.8%) | 298 (28.2%) | 399 (74.0%) | 140 (26.0%) | 359 (69.4%) | 158 (30.6%) | 255 (72.4%) | 97 /27.6%) | 254 (72.4%) | 97 (27.6%) | 249 (70.5%) | 104 (29.5%) |
| 1-5 y | 566 (77.3%) | 166 (22.7%) | 280 (78.2%) | 78 (21.8%) | 286 (76.5%) | 88 (23.5%) | 178 (77.7%) | 51 (22.3%) | 189 (78.4%) | 52 (21.6%) | 199 (76.0%) | 63 (24.0%) |
| 6 y or more | 976 (66.0%) | 503 (34.0%) | 502 (68.6%) | 230 (31.4%) | 474 (63.5%) | 273 (36.5%) | 343 (68.7%) | 156 (31.3%) | 339 (65.2%) | 181 (34.8%) | 294 (63.9%) | 166 (36.1%) |
| Child sex (girl) | 1199§ (74.6%) | 409 (25.4%) | 618§ (76.5%) | 190 (23.5%) | 581§ (72.5%) | 219 (27.4%) | 399† (75.9%) | 127 (24.1%) | 406* (73.7%) | 145 (26.3%) | 394§ (74.2%) | 137 (25.8%) |
| Child sex (boy) | 1101§ (66.4%) | 558 (33.6%) | 563§ (68.6%) | 258 (31.4%) | 538§ (64.2%) | 300 (35.8%) | 377† (68.1%) | 177 (31.9%) | 376* (67.0%) | 185 (33.0%) | 348§ (64.0%) | 196 (36.0%) |
| Length of gestation (weeks) | 39.3 (1.8) | 39.3 (2.0) | 39.3 (1.8) | 39.3 (2.0) | 39.2 (1.8) | 39.3 (2.0) | 39.3 (1.8) | 39.4 (1.9) | 39.3 (1.8) | 39.4 (2.1) | 39.2 (1.9) | 39.2 (2.0) |
| Birth weight (g) | 2691 (393) | 2701 (450) | 2699 (389) | 2707 (433) | 2682 (397) | 2696 (465) | 2690 (384) | 2737 (440) | 2696 (378) | 2676 (452) | 2686 (417) | 2693 (458) |
| Birth weight z score | -1.25 (0.92) | -1.24 (0.97) | -1.23 (0.90) | -1.23 (0.93) | -1.27 (0.93) | -1.26 (1.00) | -1.27 (0.91) | -1.23 (0.94) | -1.26 (0.91) | -1.29 (0.95) | -1.22 (0.92) | -1.22 (1.02) |

*p<0.05; †p<0.01; §p<0.001. P values comparing participants with non-participants are based on χ^2^ or t tests. For simplicity, they are marked only in the participant column.

The numbers are n (%) or mean (SD).

Fe30F, 30 mg of iron and 400 µg folic acid; Fe60F, 60 mg of iron and 400 µg folic acid; MMS, multiple micronutrient supplementation; SES, socio-economic status

Supplementary table 2. Correlations of cardiometabolic markers with height and body mass index.

|  | Height z score | BMI z score |
| --- | --- | --- |
| Total cholesterol (mmol/l) | -0.06^2^ | 0.11^3^ |
| LDL choleterol (mmol/l) | -0.03 | 0.14^3^ |
| HDL cholesterol (mmol/l) | -0.09^3^ | -0.24^3^ |
| Triglycerides (mmol/l) | -0.01 | 0.19 |
| Apolipoprotein A1 (g/l) | -0.11^3^ | -0.14^3^ |
| Apolipoprotein B (g/l) | -0.04 | 0.18^3^ |
| Glucose (mmol/l) | 0.02 | 0.00 |
| Insulin (pmol/l) | 0.09^3^ | 0.33^3^ |
| HOMA-IR | 0.09^3^ | 0.33^3^ |
| Sex hormone binding globulin (nmol/l) | -0.26^3^ | -0.55^3^ |
| Kreatinine (µmol/l) | 0.32^3^ | 0.26^3^ |
| Cystatin C (mg/l) | -0.01 | 0.04 |
| CRP (mg/l) | 0.02 | 0.26^3^ |
| Systolic blood pressure (mmHg)^1^ | 0.21^3^ | 0.37^3^ |
| Diastolic blood pressure (mmHg)^1^ | 0.14^3^ | 0.22^3^ |
| Cardiometabolic risk score^1^ | 0.18^3^ | 0.61^3^ |

The numbers are partial correlation coefficients with log-transformed cardiometabolic markers adjusted for sex and current age. Coefficients for insulin and HOMA-IR are in addition adjusted for storage time of insulin samples.

HDL, high-density lipoprotein; HOMA-IR, Homeostasis Model Assessment equation index for insulin resistance equation: (fasting insulin in mU/L × fasting glucose in mmol/L) ÷ 22.5; LDL, low-density lipoprotein

^1^No logarithmic transformation; ^2^p<0.05; ^3^p<0.001

Supplementary table 3. Mean differences in body size, cardiometabolic markers and blood pressure, and odds ratios for stunting according to early vs usual invitation to food supplementation, and according to multiple micronutrient supplementation (MMS). In addition to models shown in Table 3 additional adjustment models

|  | **Early invitation compared with usual invitation, crude mean difference (95% CI)** | **Early invitation compared with usual invitation, adjusted^1^ mean difference (95% CI)** | **Early invitation compared with usual invitation, adjusted^2^ mean difference (95% CI)** | **MMS compared with Fe60F, crude mean difference (95% CI)** | **MMS compared with Fe60F, adjusted^1^ mean difference (95% CI)** | **MMS compared with Fe60F, adjusted^2^ mean difference (95% CI)** |
| --- | --- | --- | --- | --- | --- | --- |
| Height z score | 0.08 (0.01, 0.16) | 0.09 (0.02, 0.16) | 0.09 (0.02, 0.15) | -0.01 (-0.10. 0.07) | -0.01 (-0.10, 0.07) | -0.01 (-0.09, 0.07) |
| Stunted (odds ratio) | 0.77 (0.63, 0.95) | 0.74 (0.60, 0.92) | 0.75 (0.60,0.93) | 1.14 (0.98, 1.45) | 1.13 (0.87, 1.47) | 1.12 (0.86,1.48) |
| BMI z score | 0.02 (-0.08, 0.13) | 0.04 (-0.06, 0.14) | 0.05 (-0.05, 0.15) | -0.02 (-0.15, 0.10) | -0.02 (-0.14, 0.10) | -0.02 (-0.13, 0.10) |
| Total cholesterol (mmol/l) | 0.2% (-1.3%, 1.7%) | 0.2% (-1.3%, 1.7%) | 0.2% (-1.3%, 1.7%) | -0.3% (-2.1%, 1.6%) | -0.3% (-2.1%, 1.5%) | -0.3% (-2.1%, 1.5%) |
| LDL choleterol (mmol/l) | -0.8% (-2.9%, 1.4%) | -0.7% (-2.8%, 1.5%) | -0.8% (-2.8%, 1.4%) | -0.3% (-2.9%, 2.4%) | -0.3% (-2.9%, 2.3%) | -0.3% (-2.8%, 2.3%) |
| HDL cholesterol (mmol/l) | 0.9% (-0.9%, 2.7%) | 0.9% (-0.9%, 2.7%) | 1.2% (-0.5%, 3.0%) | -2.0% (-4.1%, 0.2%) | -2.0% (-4.1%, 0.1%) | -2.0% (-4.1%, 0.0%) |
| Triglycerides (mmol/l) | 1.8 % (-1.6%, 5.3%) | 1.5% (-1.8%, 5.0%) | 2.2% (-0.5%, 4.5%) | 1.4% (-2.6%, 5.5%) | 1.4% (-2.5%, 5.5%) | 1.7% (-2.2%, 5.7%) |
| Apolipoprotein A1 (g/l) | 0.7% (-0.6%, 2.0%) | 0.7% (-0.6%, 2.0%) | 0.9% (-0.4%, 2.1%) | -1.0% (-2.5%, 0.5%) | -1.0% (-2.5%, 0.5%) | -1.0% (-2.5%, 0.5%) |
| Apolipoprotein B (g/l) | -0.8% (-2.6%, 1.0%) | -0.7% (-2.4%, 1.0%) | -0.9% (-2.6%, 0.8%) | 0.2% (-2.0%, 2.4%) | 0.2% (-1.9%, 2.3%) | 0.2% (-1.9%, 2.3%) |
| Glucose (mmol/l) | 0.7% (-0.1%, 1.4%) | 0.6% (-0.1%, 1.4%) | 0.6% (-0.2%, 1.3%) | -0.1% (-1.0%, 0.8%) | -0.1% (-1.0%, 0.8%) | -0.1% (-1.0%, 0.8%) |
| Insulin (pmol/l)^2^ | 4.2% (-1.8%, 10.6%) | 4.3% (-1.7%, 10.6%) | 3.0% (-2.6%, 8.9%) | -0.2% (-7.1%, 7.2%) | -0.2% (-7.0%, 7.0%) | 0.1% (-6.4%, 7.0%) |
| HOMA-IR^2^ | 4.4% (-1.7%, 10.8%) | 4.4% (-1.6%, 10.7%) | 3.1% (-2.5%, 9.0%) | -0.2% (-7.2%, 7.2%) | -0.2% (-7.0%, 7.1%) | 0.1% (-6.4%, 7.0%) |
| Sex hormone binding globulin (nmol/l) | -0.6% (-4.9%, 3.8%) | -1.6% (-5.5%, 2.5%) | -0.5% (-4.0%, 3.1%) | 0.3% (-4.9%, 5.7%) | 0.2% (-4.7%, 5.3%) | -0.1% (-4.3%, 4.2%) |
| Kreatnine (µmol/l) | -0.7% (-2.3%, 0.9%) | -0.5% (-1.8%, 0.9%) | -0.5% (-1.9%, 0.8%) | -0.6% (-2.5%, 1.3%) | -0.6% (-2.2%, 1.1%) | -0.5% (-2.1%, 1.1%) |
| Cystatin C (mg/l) | 0.3% (-1.1%, 1.7%) | 0.3% (-0.7%, 1.4%) | 0.4% (-0.7%, 1.4%) | 0.1% (-1.6%, 1.8%) | 0.0% (-1.2%, 1.3%) | 0.0% (-1.3%, 1.3%) |
| CRP (mg/l) | -9.6% (-19.4%. 1.3%) | -8.3% (-18.2%, 2.7%) | -9.6% (-19.0%, 0.9%) | 1.2% (-12.0%, 16.4%) | 1.8% (-11.5%, 17.1%) | 1.4% (-11.4%, 16.1%) |
| Systolic blood pressure (mmHg) | 0.4 (-0.2, 1.1) | 0.4 (-0.2, 1.2) | 0.4 (-0.2, 1.0) | -0.5 (-1.3, 0.3) | -0.6 (-1.3, 0.2) | -0.5 (-1.3, 0.2) |
| Diastolic blood pressure (mmHg) | 0.3 (-0.3, 0.9) | 0.3 (-0.3, 0.9) | 0.3 (-0.3, 0.9) | -0.4 (-1.1, 0.4) | -0.4 (-1.1, 0.4) | -0.4 (-1.1, 0.4) |
| Cardiometabolic risk score | 0.035 (-0.016, 0.086) | 0.037 (-0.014, 0.088) | 0.020 (-0.020, 0.060) | 0.005 (-0.056, 0.066) | 0.006 (-0.055, 0.067) | 0.011 (-0.037, 0.059) |

HDL, high-density lipoprotein; HOMA-IR, Homeostasis Model Assessment equation index for insulin resistance equation: (fasting insulin in mU/L × fasting glucose in mmol/L) ÷ 22.5; LDL, low-density lipoprotein

^1^Adjusted for sex, age and maternal variables during pregnancy: asset score, maternal height and maternal age.

^2^Adjusted for sex, age, BMI and maternal variables during pregnancy: asset score, maternal height, age, primiparity, education, height, BMI.

^3^Adjusted in addition for insulin sample storage time.
